# Blood transcriptional biomarkers of acute viral infection for detection of pre-symptomatic SARS-CoV-2 infection: a nested, case-control diagnostic accuracy study

**DOI:** 10.1016/S2666-5247(21)00146-4

**Published:** 2021-10

**Authors:** Rishi K Gupta, Joshua Rosenheim, Lucy C Bell, Aneesh Chandran, Jose A Guerra-Assuncao, Gabriele Pollara, Matthew Whelan, Jessica Artico, George Joy, Hibba Kurdi, Daniel M Altmann, Rosemary J Boyton, Mala K Maini, Aine McKnight, Jonathan Lambourne, Teresa Cutino-Moguel, Charlotte Manisty, Thomas A Treibel, James C Moon, Benjamin M Chain, Mahdad Noursadeghi, COVIDsortium Investigators, Hakam Abbass, Aderonke Abiodun, Mashael Alfarih, Zoe Alldis, Daniel M Altmann, Oliver E Amin, Mervyn Andiapen, Jessica Artico, João B Augusto, Georgiana L Baca, Sasha NL Bailey, Anish N Bhuva, Alex Boulter, Ruth Bowles, Rosemary J Boyton, Olivia V Bracken, Ben O'Brien, Tim Brooks, Natalie Bullock, David K Butler, Gabriella Captur, Nicola Champion, Carmen Chan, Aneesh Chandran, David Collier, Jorge Couto de Sousa, Xose Couto-Parada, Teresa Cutino-Moguel, Rhodri H Davies, Brooke Douglas, Cecilia Di Genova, Keenan Dieobi-Anene, Mariana O Diniz, Anaya Ellis, Karen Feehan, Malcolm Finlay, Marianna Fontana, Nasim Forooghi, Celia Gaier, Joseph M Gibbons, Derek Gilroy, Matt Hamblin, Gabrielle Harker, Jacqueline Hewson, Lauren M Hickling, Aroon D Hingorani, Lee Howes, Alun Hughes, Gemma Hughes, Rebecca Hughes, Ivie Itua, Victor Jardim, Wing-Yiu Jason Lee, Melaniepetra Jensen, Jessica Jones, Meleri Jones, George Joy, Vikas Kapil, Hibba Kurdi, Jonathan Lambourne, Kai-Min Lin, Sarah Louth, Mala K Maini, Vineela Mandadapu, Charlotte Manisty,, Áine McKnight, Katia Menacho, Celina Mfuko, Oliver Mitchelmore, Christopher Moon, James C Moon,, Diana Munoz Sandoval, Sam M Murray, Mahdad Noursadeghi, Ashley Otter, Corinna Pade, Susana Palma, Ruth Parker, Kush Patel, Babita Pawarova, Steffen E Petersen, Brian Piniera, Franziska P Pieper, Daniel Pope, Maria Prossora, Lisa Rannigan, Alicja Rapala, Catherine J Reynolds, Amy Richards, Matthew Robathan, Joshua Rosenheim, Genine Sambile, Nathalie M Schmidt, Amanda Semper, Andreas Seraphim, Mihaela Simion, Angelique Smit, Michelle Sugimoto, Leo Swadling, Stephen Taylor, Nigel Temperton, Stephen Thomas, George D Thornton, Thomas A Treibel, Art Tucker, Jessry Veerapen, Mohit Vijayakumar, Sophie Welch, Theresa Wodehouse, Lucinda Wynne, Dan Zahedi

**Affiliations:** a Institute of Global Health, University College London, London, UK; b Division of Infection and Immunity, University College London, London, UK; c Institute of Cardiovascular Sciences, University College London, London, UK; d Barts Heart Centre, St Bartholomew's Hospital, Barts Health NHS Trust, London, UK; e Department of Infection, St Bartholomew's Hospital, Barts Health NHS Trust, London, UK; f Department of Virology, St Bartholomew's Hospital, Barts Health NHS Trust, London, UK; g Department of Immunology and Inflammation, Imperial College London, London, UK; h Lung Division, Royal Brompton & Harefield NHS Foundation Trust, London, UK; i Blizard Institute, Barts and the London School of Medicine and Dentistry, Queen Mary University of London, London, UK

## Abstract

**Background:**

We hypothesised that host-response biomarkers of viral infections might contribute to early identification of individuals infected with SARS-CoV-2, which is critical to breaking the chains of transmission. We aimed to evaluate the diagnostic accuracy of existing candidate whole-blood transcriptomic signatures for viral infection to predict positivity of nasopharyngeal SARS-CoV-2 PCR testing.

**Methods:**

We did a nested case-control diagnostic accuracy study among a prospective cohort of health-care workers (aged ≥18 years) at St Bartholomew's Hospital (London, UK) undergoing weekly blood and nasopharyngeal swab sampling for whole-blood RNA sequencing and SARS-CoV-2 PCR testing, when fit to attend work. We identified candidate blood transcriptomic signatures for viral infection through a systematic literature search. We searched MEDLINE for articles published between database inception and Oct 12, 2020, using comprehensive MeSH and keyword terms for “viral infection”, “transcriptome”, “biomarker”, and “blood”. We reconstructed signature scores in blood RNA sequencing data and evaluated their diagnostic accuracy for contemporaneous SARS-CoV-2 infection, compared with the gold standard of SARS-CoV-2 PCR testing, by quantifying the area under the receiver operating characteristic curve (AUROC), sensitivities, and specificities at a standardised Z score of at least 2 based on the distribution of signature scores in test-negative controls. We used pairwise DeLong tests compared with the most discriminating signature to identify the subset of best performing biomarkers. We evaluated associations between signature expression, viral load (using PCR cycle thresholds), and symptom status visually and using Spearman rank correlation. The primary outcome was the AUROC for discriminating between samples from participants who tested negative throughout the study (test-negative controls) and samples from participants with PCR-confirmed SARS-CoV-2 infection (test-positive participants) during their first week of PCR positivity.

**Findings:**

We identified 20 candidate blood transcriptomic signatures of viral infection from 18 studies and evaluated their accuracy among 169 blood RNA samples from 96 participants over 24 weeks. Participants were recruited between March 23 and March 31, 2020. 114 samples were from 41 participants with SARS-CoV-2 infection, and 55 samples were from 55 test-negative controls. The median age of participants was 36 years (IQR 27–47) and 69 (72%) of 96 were women. Signatures had little overlap of component genes, but were mostly correlated as components of type I interferon responses. A single blood transcript for *IFI27* provided the highest accuracy for discriminating between test-negative controls and test-positive individuals at the time of their first positive SARS-CoV-2 PCR result, with AUROC of 0·95 (95% CI 0·91–0·99), sensitivity 0·84 (0·70–0·93), and specificity 0·95 (0·85–0·98) at a predefined threshold (Z score >2). The transcript performed equally well in individuals with and without symptoms. Three other candidate signatures (including two to 48 transcripts) had statistically equivalent discrimination to *IFI27* (AUROCs 0·91–0·95).

**Interpretation:**

Our findings support further urgent evaluation and development of blood *IFI27* transcripts as a biomarker for early phase SARS-CoV-2 infection for screening individuals at high risk of infection, such as contacts of index cases, to facilitate early case isolation and early use of antiviral treatments as they emerge.

**Funding:**

Barts Charity, Wellcome Trust, and National Institute of Health Research.

## Introduction

Rapid and accurate testing is central to effective public health responses to COVID-19. Infectivity, measured by SARS-CoV-2 titres in the upper respiratory tract, peaks during the first week of symptoms.^1^ Early case detection and subsequent rapid isolation of index cases, alongside contact tracing and quarantine, are key interventions to interrupt onward transmission. Because some individuals with SARS-CoV-2 shed virus while asymptomatic or pauci-symptomatic,^2^, ^3^ there is also global interest in screening tests for at-risk individuals who do not fulfil case definition criteria and in mass testing for early case detection among the general population regardless of symptoms.^4^

**Research in context**

**Evidence before this study**

We searched MEDLINE for studies published between database inception and Oct 12, 2020, using comprehensive search terms for “biomarker”, “viral infection”, “blood”, and “transcriptome”. We did not restrict by language or study type. Full details of our search strategy are in appendix 1 (p 5). Our search returned 1150 studies, 61 of which were relevant to this topic. Early case detection and isolation are key interventions to interrupt transmission of SARS-CoV-2 infection. A range of blood transcriptomic biomarkers have been proposed for the detection of viral infections. However, the diagnostic accuracy of these candidate signatures has not been previously evaluated for early SARS-CoV-2 infection.

**Added value of this study**

In this prospective diagnostic accuracy study within a cohort of health-care workers with paired serial sampling of blood (for RNA sequencing and SARS-CoV-2 serology) and nasopharyngeal swabs (for viral PCR), we identified 20 previously proposed blood transcriptomic signatures for viral infection through our systematic literature search, and calculated signature scores for each sample according to original descriptions. Four signatures reflecting type I interferon signalling (including a single transcript *IFI27*) discriminated between test-negative controls and contemporaneous SARS-CoV-2 PCR positivity, with statistically equivalent performance. Using a pre-specified diagnostic threshold, *IFI27* achieved sensitivity of 84% (95% CI 70–93) and specificity of 95% (85–98) for contemporaneous PCR positivity, with sensitivity at 40% (17–69) 1 week before first positive PCR test.

**Implications of all the available evidence**

Blood transcriptomic biomarkers for viral infection, including *IFI27*, reflect underlying type I interferon responses and detect early SARS-CoV-2 infection with high accuracy. If these biomarkers are included in scalable point-of-care tests for SARS-CoV-2, they could facilitate early case detection and contact investigation.

Effective screening tests must be accurate and reliable.^5^ Current tools, such as lateral flow assays for SARS-CoV-2 antigens, have inadequate sensitivity to effectively rule out active infection and might have low value for contact and general population screening.^6^ RT-PCR tests, which identify viral RNA, are the current gold standard for diagnosis of SARS-CoV-2 infection, but pose various challenges including test speed and the requirement of a skilled laboratory operator.^7^ Loop-mediated isothermal amplification (referred to as LAMP) assays have quicker test speeds than RT-PCR timings, but with an associated reduction in sensitivity.^8^ All detection tests currently available for SARS-CoV-2 rely on swabbing of nasopharyngeal or oropharyngeal mucosa, or both, the effectiveness of which is operator-dependent and prone to sampling variability. Although positive SARS-CoV-2 test results are useful in clinical management and infection control settings, negative results—in the context of high pre-test probability of a positive finding—mean that the current tests cannot be used to rule out infection effectively.^9^

There is a clear need to expand the portfolio of tests available for the identification of SARS-CoV-2 infection, for both screening and diagnostic purposes. Measurement of the host response, as opposed to viral targets, is one potential diagnostic strategy. Numerous studies have demonstrated whole-blood transcriptional perturbation during other acute viral infections, such as influenza, rhinovirus, and respiratory syncitial virus.^10^, ^11^, ^12^, ^13^ A range of blood transcriptomic signatures have, therefore, been proposed as candidate diagnostic biomarkers for various purposes, including discrimination of viral from bacterial infection or no infection,^10^, ^11^, ^12^, ^13^, ^14^, ^15^, ^16^, ^17^, ^18^, ^19^, ^20^, ^21^ diagnosis of pre-symptomatic viral infection in known contacts,^22^ diagnosis of specific viral infections,^23^, ^24^ or prediction of severity.^25^ These signatures have not yet been evaluated for early diagnosis of pre-symptomatic or mild SARS-CoV-2 infection. We aimed to systematically evaluate the potential for existing candidate whole-blood transcriptomic signatures of viral infection to predict nasopharyngeal SARS-CoV-2 PCR test positivity in health-care workers undergoing weekly testing with paired blood RNA sampling.

## Methods

### Study design and participants

This prospective diagnostic accuracy study, with a case-control design, was nested within our COVIDsortium observational cohort study in health-care workers (NCT04318314). Participant screening, study design, sample collection, and sample processing have been described previously.^26^, ^27^, ^28^ Briefly, health-care workers (aged ≥18 years) were recruited at St Bartholomew's Hospital, London, UK in the first week of lockdown in the UK (between March 23 and March 31, 2020). Participants were assessed weekly using a questionnaire and paired serial sampling of blood (for RNA sequencing and SARS-CoV-2 serology) and nasopharyngeal swabs (for viral PCR), for up to 16 weeks when they were fit enough to attend work (according to Barts Health NHS Trust policy) at each visit, with further follow-up samples collected at week 24. The questionnaire included questions about symptom burden; symptoms were classified as case defining (fever, new continuous dry cough, or a new loss of taste or smell [anosmia]), non-case defining (specific symptoms other than case-defining symptoms, or unspecified symptoms), or asymptomatic (no symptoms reported).

Participants who were not available for a particular visit (eg, due to shift pattern, annual leave redeployment, self-isolation, or illness) resumed follow-up on their return to work.

We used the Roche cobas 8800 diagnostic test platform (Burgess Hill, UK) as the standard reference for PCR confirmation of SARS-CoV-2 infection, with a cycle threshold of 40. Participants with available blood RNA samples who had PCR-confirmed SARS-CoV-2 infection at any timepoint during the study were included in the test-positive group (ie, cases) and their blood RNA samples were sequenced. A subset of consecutively recruited participants without evidence of SARS-CoV-2 infection on nasopharyngeal swabs and who remained seronegative by both Euroimmun anti-S1 spike protein and Roche anti-nucleocapsid protein throughout follow-up were included in the test-negative control group; only their baseline blood RNA samples were sequenced.

The study was approved by the South Central–Oxford A Research Ethics Committee (reference 20/SC/0149), and the study was done in accordance with the principles of the Declaration of Helsinki and Good Clinical Practice. All participants provided written informed consent.

### Systematic search for candidate transcriptional signatures

We did a systematic literature search of peer-reviewed publications to identify concise blood transcriptional signatures discovered or applied with a primary objective of diagnosis or assessment of severity of viral infection from human whole-blood or peripheral blood mononuclear cell samples. We searched MEDLINE for articles published between database inception and Oct 12, 2020, using comprehensive MeSH and keyword terms for “viral infection”, “transcriptome”, “biomarker”, and “blood”. Our search had no language restrictions. Our full search strategy is in appendix 1 (p 5). We identified additional studies in reference lists and from expert consultation. Titles and abstracts were initially screened by two independent reviewers (RKG and LCB); full-text reviews were done for shortlisted articles to determine eligibility and conflicts were resolved through discussion and arbitration by a third reviewer (MN) where required. We focused on concise signatures that might be more amenable to translation to diagnostic tests; we defined concise signatures as any signature discovered using a defined approach to feature selection to reduce the number of constituent genes, as previously described.^29^ We required that gene names that comprised the signature were publicly available, along with the corresponding signature equation or model coefficients, and that the signature was validated in at least one independent test or validation set to prioritise signatures discovered from higher quality studies. Where multiple signatures were discovered for the same intended purpose and from the same discovery cohort, we included the signature with highest discrimination (as defined by the area under the receiver operating characteristic curve [AUROC]) in the validation data, or the signature with the fewest number of genes when accuracy was equivalent.

For each signature that met our eligibility criteria, we extracted constituent genes, modelling approaches, and coefficients to enable independent reconstruction of signature scores. Extraction was done by one reviewer (RKG) and was verified by a second reviewer (LCB).

We refer to RNA signatures by combining the first author's name of the corresponding publication as a prefix, with the number of constituent genes as a suffix; except for single-gene signatures, which are referred to by the gene name.

### Blood RNA sequencing

For the positive-test group, we included all available RNA samples within 3 weeks of first positive SARS-CoV-2 PCR test, and convalescent samples at week 24 of follow-up for a subset of participants with available samples. For the control group, we included baseline samples only. Genome-wide mRNA sequencing was done as previously described^30^ using the Kappa Hyperprep kit (Roche; Burgess Hill, UK) to generate complementary DNA libraries sequenced on the Illumina Nextseq platform using the Nextseq 500/550 High Output 75 cycle kit (Illumina; Cambridge, UK) according to manufacturers' instructions, giving a median of 26 million (range 19·8–32·4) 41-base pair paired-end reads per sample. We mapped RNAseq data to the reference transcriptome (Ensembl Human GRCh38 release 100) using Kallisto (version 0.46.1).^31^ The transcript-level output counts and transcripts per million values were summed on gene level and annotated with Ensembl gene ID, gene name, and gene biotype using the tximport (version 1.20.0) and biomaRt (version 2.48.0) Bioconductor packages in R.^32^, ^33^

### Outcomes

The primary outcome was the AUROC for discriminating between control samples and samples from participants with PCR-confirmed SARS-CoV-2 infection during their first week of PCR test positivity. The secondary outcome was the AUROC for discriminating between control samples and samples from participants with PCR-confirmed SARS-CoV-2 infections in the week before first positive PCR test.

### Statistical analysis

The statistical power in our primary analysis is provided for a range of AUROCs 0·5–1·0 (appendix 1, p 3). Our sample size provided more than 90% power to discriminate between test-positive cases and test-negative controls with an AUROC of at least 0·7. For each eligible signature, we reconstructed signature scores as per the original authors' descriptions. For logistic and probit regression models, we calculated scores on the linear predictor scale by summing the expression of each constituent gene multiplied by its coefficient. Scores for each signature were standardised to Z scores using the mean and SD among the control group. We multiplied scores that were designed to decrease in viral infection by −1 to ensure that higher scores were associated with higher risk of viral infection across all candidate signatures.

We calculated AUROCs and corresponding sensitivities and specificities with 95% CIs for each signature for the primary and secondary outcomes using prespecified cutoffs based on two SDs above the mean value of the controls (referred to as Z2) as previously described.^29^ To identify the subset of best performing signatures, we used pairwise DeLong tests for the signature with the highest AUROC for the primary outcome (or most parsimonious in the event of equal performance), with adjustment for multiple testing using a Benjamini-Hochberg correction.^34^ Signatures were considered to have statistically inferior accuracy to the best performing signature if the adjusted value was p<0·05. We evaluated associations between the best performing signatures and SARS-CoV-2 PCR cycle thresholds among people with contemporaneous PCR test positivity visually using scatterplots and Spearman rank correlation (reported as *r*).

We used Ingenuity Pathway Analysis (Qiagen; Venlo, Netherlands) for upstream analysis of transcriptional regulation of the constituent genes in the candidate signatures. Findings were visualised as network diagrams in Gephi (version 0.9.2), depicting all statistically overrepresented molecules that were predicted to be upstream of at least two target genes, as previously described.^29^ We evaluated pairwise Spearman rank and Jaccard indices between each candidate signature to quantify correlations and proportions of intersecting genes between signatures.

We did sensitivity analyses to assess the effect of various factors on our findings. First, we recalculated discrimination (AUROC) for the primary outcome, excluding participants with positive SARS-CoV-2 nasopharyngeal swabs who reported contemporaneous case-defining symptoms at the time of sampling to evaluate diagnostic accuracy for people without case-defining symptoms. Second, we assessed the discrimination of the best performing signatures when using peak signatures scores for each participant during follow-up. Finally, we used a multivariable linear regression model to evaluate whether age, sex, or presence of concurrent case-defining symptoms were associated with expression of the best performing signature, after adjustment for the SARS-CoV-2 PCR cycle threshold.

We used R (version 3.6.3) for all statistical analyses.

### Role of the funding source

The funders of the study had no role in study design, data collection, data analysis, data interpretation, or writing of the report.

## Results

**Figure 1:**
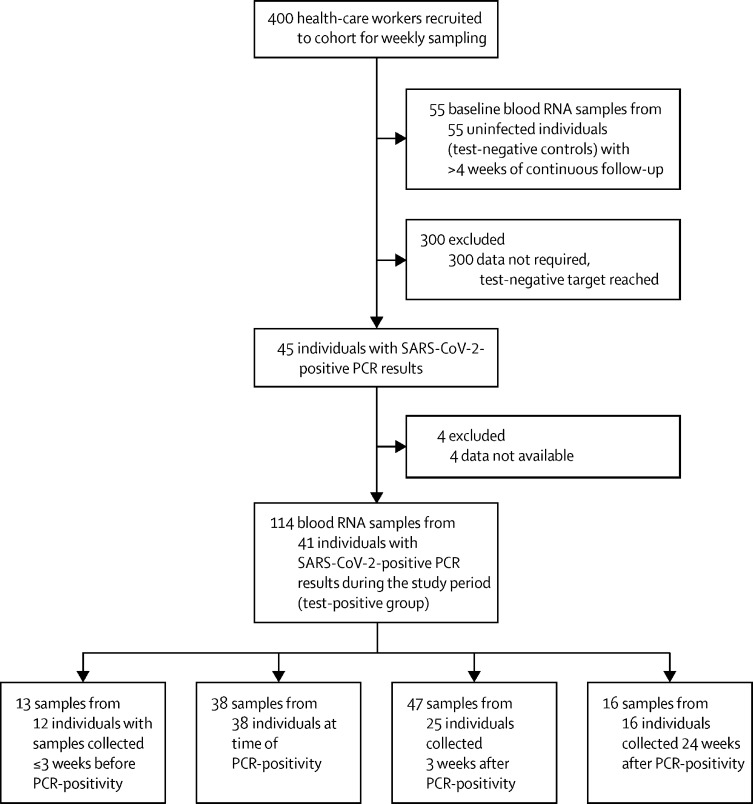
Study profile

Between March 23 and March 31, 2020, we recruited health-care workers for this analysis. We included 169 blood RNA samples from 96 participants in a nested case-control study (figure 1) derived from an observational health-care worker cohort.^26^, ^27^, ^28^ Of these, 114 samples (including 16 convalescent samples collected 24 weeks after infection) were from 41 participants with SARS-CoV-2 infection during the study period, and 55 samples were from 55 test-negative controls.

The median age of participants was 36 years (IQR 27–47) and 69 (72%) of 96 were female; White (66 participants [69%]) was the most common ethnicity, followed by Asian (18 [19%]) and Black (six [6%]). Full baseline characteristics and the number of included blood RNA samples per participant are listed in appendix 1 (p 6). 32 (78%) of 41 test-positive participants denied having any disease defining symptoms at the time of their SARS-CoV-2-positive PCR test, whilst nine (22%) of 41 described having one or more of cough, fever, or anosmia. A further 22 participants developed symptoms during subsequent follow-up. All symptomatic participants had mild disease. None of the control participants had alternative diagnoses.

Our systematic literature search found 1150 titles and abstracts; 61 studies were shortlisted for full-text review. 18 studies, describing 20 distinct transcriptional signatures for viral infection, met the eligibility criteria for inclusion in the final analysis (table 1; appendix 1 p 2). Signatures comprised between two and 48 component genes and were discovered in various populations, including children and adults with acute viral infections, adults experimentally challenged with viruses (such as influenza, respiratory syncytial virus [RSV], and rhinovirus). 12 (60%) of 20 signatures were discovered with the objective of discriminating viral infection from bacterial or other inflammatory presentations.^10^, ^11^, ^12^, ^13^, ^14^, ^15^, ^16^, ^17^, ^18^, ^19^, ^20^, ^21^ Three signatures aimed to discriminate between healthy individuals and those with viral infection^37^, ^39^ and two were discovered with a specific objective of diagnosing influenza infection.^23^, ^24^ One signature aimed to predict the severity of RSV infection in children.^25^ One study evaluated a pre-existing signature with the aim of identifying pre-symptomatic viral infection in individuals who were close contacts of index cases with acute viral respiratory tract infections.^22^

**Table 1:** Characteristics of whole-blood RNA signatures for viral infection included in analysis

	Model*	**Discovery populations**	**Discovery settings**	**Discovery approach**	**Validation populations**	**Intended application**
AndresTerre11^23^	Geometric mean of all genes (influenza meta-signature)	Five cohorts of children and adults with influenza; adults challenged with influenza; and adults with bacterial pneumonia	UK, USA, and Australia	Differential expression followed by leave-one-cohort-out strategy and filtering for heterogeneity of effect size, using genome-wide data	Eight cohorts of children or adults with influenza or bacterial infection; adults challenged with influenza; and adults vaccinated against influenza	Influenza *vs* bacterial or other viral infection
Henrickson16^24^	Difference in geometric means between upregulated and downregulated genes (influenza paediatric signature score)	Four cohorts of children with influenza-like illness	USA	Meta-analysis and leave-one-out strategy to identify common genes using genome-wide data	Two cohorts of children or adults with influenza	Influenza infection *vs* healthy
Herberg2^10^	Disease risk score†	Children with viral or bacterial infection	UK, USA, and Spain	Elastic net followed by forward selection–partial least squares, using significantly differentially expressed transcripts	Children with bacterial or viral infection, inflammatory disease, or indeterminate diagnosis	Viral *vs* bacterial infection in febrile children
*IFI44L*^14^	NA	Children with viral or bacterial infection^10^	UK, USA, and Spain	Elastic net followed by forward selection–partial least squares, using significantly differentially expressed transcripts	Children with bacterial or viral infection	Viral *vs* bacterial infection in febrile children
*IFIT3*;*RSAD2*^22^‡	NA	Three cohorts of adults challenged with rhinovirus, influenza, or RSV^35^	UK and USA	Sparse latent factor regression analysis on genome-wide data^35^ followed by regularised logistic regression on the resulting 30-gene signature	Close contacts of students with acute upper respiratory viral infections	Pre-symptomatic viral infection *vs* healthy
Lopez7^15^	Sum of weighted gene expression values (bacterial *vs* viral classifier)	Children and adults with viral, bacterial, or non-infectious acute respiratory illness^19^	USA	Support vector machine analysis using genome-wide data	Children with acute viral or bacterial infections^36^	Viral *vs* bacterial respiratory infection
Lydon15^11^	Logistic regression (viral classifier)§	Adolescents and adults with viral, bacterial, or non-infectious acute respiratory illness	USA	LASSO regression analysis using 87 selected target genes from previously derived signatures^19^, ^21^	Patients with viral or bacterial co-infection or suspected bacterial infection	Viral *vs* bacterial respiratory infection
*MX1*^37^	NA	NA	NA	Preselected due to biological plausibility	Adults challenged with the live yellow fever virus vaccine	Viral infection *vs* healthy
*OLFM4*^25^	NA	Children with RSV infection	The Netherlands	Differential expression and prediction analysis of microarrays classifier training using genome-wide data	A second cohort of children with RSV infection	Severity of RSV infection in children
Pennisi2^20^	Disease risk score†	Children with viral or bacterial infection^10^	UK, USA, and Spain	Elastic net followed by forward selection–partial least squares, using significantly differentially expressed transcripts,^10^ then selection of an adequately expressed transcript for use in RT-LAMP	Children with bacterial or viral infection	Viral *vs* bacterial infection in children
Sampson10^13^	Disease risk score (combined SeptiCyte score)	Eight cohorts of neonates, children, and adults with bacterial infections	UK, USA, Estonia, and Australia	Regression analysis of transcript pairs using the 6000 most highly expressed genes from each dataset	Unselected consecutive patients presenting to the emergency department with febrile illness	Viral *vs* bacterial in febrile patients
Sampson4^16^	Disease risk score (Septicyte VIRUS)	Ten cohorts of children and adults with viral infections; two cohorts of adults challenged with influenza; and two cohorts of macaques challenged with Lassa virus or lymphocytic choriomeningitis virus	USA, Brazil, Finland, and Australia	Regression analysis of transcript pairs using the 6000 most highly expressed genes from each dataset	Seven human cohorts and six non-human mammal cohorts infected or challenged with viruses across all seven of the Baltimore virus classification groups	Viral *vs* non-viral conditions
Sweeney11^17^	Difference in geometric means between upregulated and downregulated genes, multiplied by ratio of counts of positive to negative genes (Sepsis metascore)	Nine cohorts of patients with sepsis or trauma	USA, Australia, Spain, Greece, the Netherlands, Norway, Canada, and UK	Greedy forward search of 82 differentially expressed genes identified by multicohort analysis	12 cohorts of adults with viral or bacterial sepsis, or trauma	Viral or bacterial sepsis *vs* sterile inflammation
Sweeney7^12^	Difference in geometric means between upregulated and downregulated genes, multiplied by ratio of counts of positive to negative genes (bacterial or viral metascore)	Eight cohorts of children and adults with viral and bacterial infections	USA, Australia, UK	Greedy forward search of 72 differentially expressed genes identified by multicohort analysis	24 cohorts of children and adults with viral or bacterial infections, or healthy controls	Viral *vs* bacterial infection
Trouillet-Assant6^18^	Median expression of six interferon-stimulated genes (interferon score^38^)	NA	NA	Differential expression using 15 preselected interferon-stimulated genes	Febrile children with bacterial or viral infection	Viral *vs* bacterial infection in febrile children
Tsalik33^19^	Logistic regression (viral ARI classifier)§	Children and adults with viral, bacterial, or non-infectious acute respiratory illness, and healthy controls	USA	LASSO regression analysis using the 40% of microarray probes with the largest variance after batch correction	Five cohorts of children or adults with viral, bacterial, or non-infectious respiratory illness, or viral or bacterial co-infection	Viral *vs* bacterial acute respiratory illness
Yu3;*IFI27*^39^	Yu3: mean expression (non-RSV infections *vs* controls); *IFI27*: NA	Children with acute respiratory illness and a positive result for a viral infection on a nasopharyngeal swab	USA	Modified supervised principal component analysis using all expressed transcripts	Children with RSV or rhinovirus infection	Viral *vs* healthy in children
Zaas48^21^	Probit regression (viral classifier)§	Two cohorts of adults challenged with influenza A H3N2 or H1N1	USA	Elastic net using 48 selected genes comprised of: 29 derived as a signature in a previous study,^35^ seven shown to be downregulated in analysis of influenza challenge time course data,^40^ and 12 control genes	Adults presenting to the emergency department with fever and healthy controls	Viral *vs* bacterial acute respiratory illness

Log_2_-transformed transcripts per million data were used to calculate all signatures. NA=not applicable. RSV=respiratory syncytial virus. LASSO=least absolute shrinkage selector operator. RT-LAMP=reverse transcription loop-mediated isothermal amplification.

* Where applicable, the name of the signature from the original publication is indicated in brackets.

† Defined as the sum of downregulated genes subtracted from the sum of upregulated genes.

‡ Study by McClain and colleagues^22^ sought to validate a 36-transcript signature for the detection of respiratory viral infections. Model coefficients for the 36-transcript model are not provided; therefore, we included in this analysis the two best performing single transcripts from the study, since they had similar performance to the full model in the original publication.

§ Logistic and probit regression models were calculated on the linear predictor scale using model coefficients from original publications.

**Figure 2:**
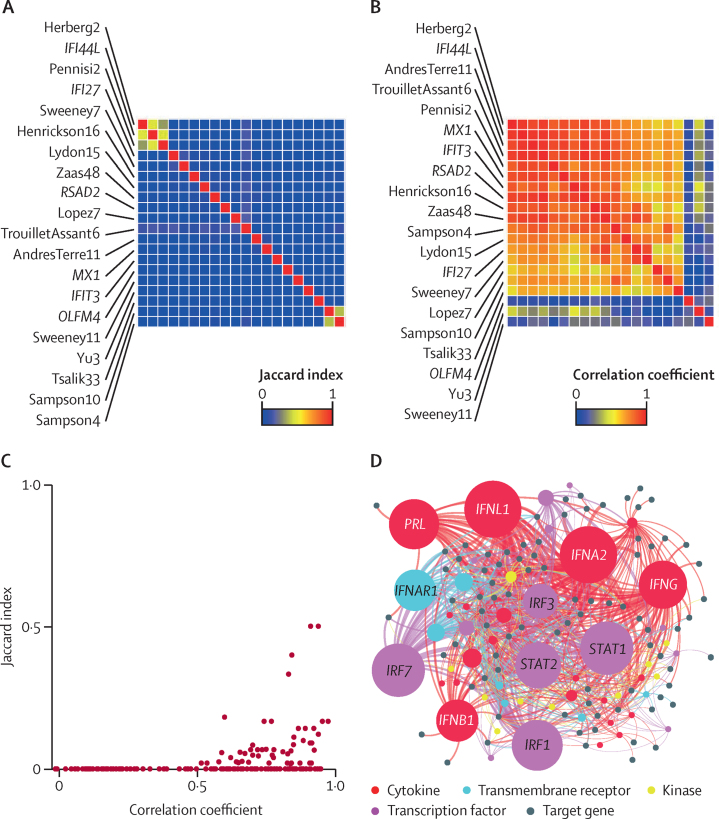
Correlation and Jaccard indices for all eligible RNA signatures for viral infection (A) Jaccard index intersect of constituent genes for all pairs of signatures clustered by Euclidean distance, indicating the proportion of the gene list that overlap in each pairwise comparison of signatures. The order of row labels for individual signatures is mirrored in the columns of the heatmap. (B) Spearman rank correlation coefficients for all pairs of signatures clustered by 1 – Spearman rank distance. The order of row labels for individual signatures is mirrored in the columns of the heatmap. (C) Relationship between pairwise Jaccard indices and Spearman rank correlation coefficients. (D) Network plot of significantly enriched predicted upstream regulators by cytokine, transmembrane receptors, kinase, and transcription factors of all constituent genes in any signature. The size of upstream regulator nodes is proportional to statistical enrichment. Node labels are shown for the ten most statistically enriched upstream regulators (false discovery rate <5 × 10^−17^). Full details of our upstream regulator analysis are in appendix 2.

In most instances there was little overlap between the constituent genes in each signature, but most signatures showed moderate to strong correlation, which was only partly explained by overlapping constituent genes (figure 2A–C). Bioinformatic analysis of the integrated list of constituent genes to identify upstream regulators using Ingenuity Pathway Analysis was consistent with type I interferon (IFN) regulation of these genes, which would explain the strong correlation between signatures despite limited overlap of their constituents (figure 2D; appendix 2).

**Figure 3:**
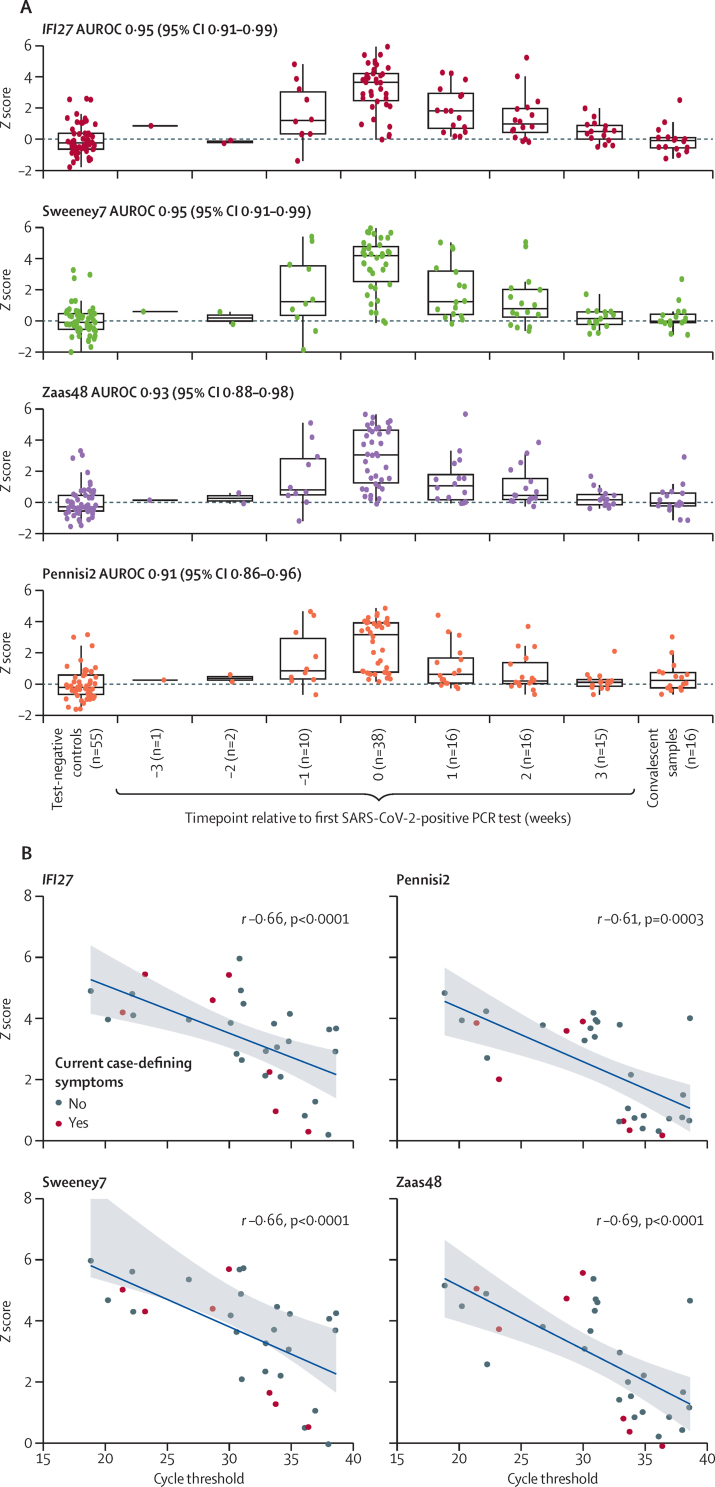
Four best performing RNA signatures for discriminating between controls and test-positive participants at the time of SARS-CoV-2-positive PCR test (A) Z scores for each RNA signature in the test-negative control group and in the test-positive control group, stratified by time relative to first SARS-CoV-2-positive PCR test. Convalescent samples were collected at study week 24. AUROC (95% CI) are for discriminating between test-negative controls and test-positive participants at the time of first SARS-CoV-2-positive PCR test (0 weeks). (B) Z scores versus contemporaneous PCR cycle threshold for SARS-CoV-2 open reading frame 1, with Spearman rank correlation coefficients. AUROC=area under the receiver operating characteristic curve.

Among all the signatures, the transcript for IFN alpha inducible protein 27 (*IFI27*) alone provided the best discrimination of contemporaneous SARS-CoV-2 infection by nasopharyngeal PCR, compared with test-negative controls, achieving an AUROC of 0·95 (95% CI 0·91–0·99; table 2). Using a prespecified Z2 cutoff based on two SDs above the mean of the test-negative control samples, *IFI27* had a sensitivity of 0·84 (95% CI 0·70–0·93) and specificity of 0·95 (0·85–0·98). Three other candidate signatures (Sweeney7, Zaas48, and Pennisi2) had statistically equivalent accuracy to *IFI27* using paired DeLong tests (AUROCs 0·91–0·95; table 2). Constituent genes for these four best performing signatures are shown in appendix 1 (p 3), of which only Pennisi2 did not include *IFI27*. Longitudinal expression of the four best performing signatures is shown in figure 3A. As a group, these peaked at the week of first positive SARS-CoV-2 PCR test and normalised at the timepoint of convalescent sampling (week 24). Scores for each of the four best performing signatures were inversely correlated with SARS-CoV-2 RT-PCR cycle thresholds, but were visually independent of current case-defining symptoms, suggesting that higher viral loads were associated with higher signature scores (figure 3B; *r* −0·61 to −0·69).

**Table 2:** Validation metrics of whole-blood RNA signatures for discrimination of participants with PCR-confirmed SARS-CoV-2 infection at first week of PCR positivity

	**AUROC**	**Sensitivity**	**Specificity**	**Adjusted p value**
*IFI27*	0·95 (0·91–0·99)	0·84 (0·70–0·93)	0·95 (0·85–0·98)	..
Sweeney7	0·95 (0·91–0·99)	0·82 (0·67–0·91)	0·95 (0·85–0·98)	0·85
Zaas48	0·93 (0·88–0·98)	0·61 (0·45–0·74)	0·95 (0·85–0·98)	0·088
Pennisi2	0·91 (0·86–0·96)	0·58 (0·42–0·72)	0·95 (0·85–0·98)	0·088
*IFI44L*	0·90 (0·84–0·96)	0·55 (0·40–0·70)	0·95 (0·85–0·98)	0·039
AndresTerre11	0·89 (0·83–0·95)	0·55 (0·40–0·70)	0·95 (0·85–0·98)	0·021
Henrickson16	0·89 (0·82–0·96)	0·55 (0·40–0·70)	0·93 (0·83–0·97)	0·0093
TrouilletAssant6	0·87 (0·80–0·94)	0·53 (0·37–0·68)	0·93 (0·83–0·97)	0·008
Lydon15	0·86 (0·79–0·94)	0·58 (0·42–0·72)	0·95 (0·85–0·98)	0·0046
Herberg2	0·84 (0·76–0·92)	0·5 (0·35–0·65)	0·93 (0·83–0·97)	0·0034
Sampson4	0·84 (0·76–0·92)	0·5 (0·35–0·65)	0·93 (0·83–0·97)	0·0027
Sampson10	0·83 (0·74–0·92)	0·5 (0·35–0·65)	0·95 (0·85–0·98)	0·0021
*RSAD2*	0·83 (0·74–0·91)	0·47 (0·32–0·63)	0·93 (0·83–0·97)	0·0021
*MX1*	0·82 (0·74–0·91)	0·45 (0·30–0·60)	0·95 (0·85–0·98)	0·0017
Tsalik33	0·79 (0·70–0·89)	0·39 (0·26–0·55)	0·98 (0·9–1·0)	0·0011
Lopez7	0·79 (0·69–0·88)	0·37 (0·23–0·53)	0·98 (0·9–1·0)	0·00080
*IFIT3*	0·75 (0·64–0·86)	0·45 (0·30–0·60)	0·93 (0·83–0·97)	0·00027
*OLFM4*	0·62 (0·51–0·74)	0·03 (0·0–0·13)	0·98 (0·9–1·0)	<0·0001
Sweeney11	0·60 (0·48–0·73)	0·16 (0·07–0·30)	0·96 (0·88–0·99)	<0·0001
Yu3	0·59 (0·47–0·71)	0·05 (0·01–0·17)	1 (0·93–1·0)	<0·0001

Data are point estimates (95% CIs). Includes 38 contemporaneous SARS-CoV-2-positive samples and 55 SARS-CoV-2-negative samples. Discrimination is shown as AUROC. Sensitivity and specificity are shown using predefined thresholds of 2 SDs above the mean of the uninfected control population (Z2). p values show pairwise comparisons to best performing signature with Benjamini-Hochberg adjustment (false discovery rate 0·05). Equivalent data for discrimination between test-negative controls and participants with SARS-CoV-2 infection 1 week before positive PCR test are in appendix 1 (p 7). AUROC=area under the receiver operating characteristic curve.

For the four best performing signatures, measurements in the week preceding the first SARS-CoV-2-positive PCR test were higher than those of controls and convalescent samples (figure 3A). AUROCs for discrimination between control samples and samples taken in the week before first SARS-CoV-2-positive PCR test showed significant discrimination for 12 of the 20 signatures assessed, but were lower than those for contemporaneous virus PCR positivity (appendix 1 p 7). Notably, *IFI27* predicted SARS-CoV-2 infection 1 week before a positive virus PCR test with an AUROC of 0·79 (95% CI 0·60–0·98). At Z2 cutoff, sensitivity was 0·40 (0·17–0·69) and specificity was 0·95 (0·85–0·98).

Exclusion of participants with contemporaneous case-defining symptoms at the time of SARS-CoV-2 infection (nine participants) did not affect the primary outcome (appendix 1 p 8). Evaluation of peak signature scores during follow-up for the four best performing signatures showed similar discrimination between the test-positive group and the test-negative control group for the primary endpoint (appendix 1 p 4). In a multivariable linear regression model, PCR cycle threshold was strongly inversely associated with the outcome of *IFI27* expression at the time of contemporaneous SARS-CoV-2-positive PCR test (appendix 1 p 9). However, there were no associations between age, sex, or current symptoms and *IFI27* scores.

## Discussion

To our knowledge, our diagnostic accuracy study is the first evaluation of host transcriptomic signatures for the detection of pre-symptomatic SARS-CoV-2 infection. By using a longitudinal blood transcriptomic dataset prospectively collected from health-care workers in London, UK, during the first wave of the COVID-19 pandemic, we systematically compared the diagnostic accuracy of 20 candidate transcriptional signatures originally discovered in a wide range of viral infection cohorts. We found that four candidate signatures—*IFI27*, Sweeney7, Zaas48, and Pennisi2—had high accuracy for discriminating between test-negative controls and test-positive participants at the time of their first SARS-CoV-2-positive PCR test (AUROCs 0·91–0·95). Three of the four signatures contained the IFN-stimulated gene *IFI27*, which was the top-performing biomarker; *IFI27* was originally discovered in a paediatric cohort^39^ to discriminate between healthy controls and those with RSV infection. Notably, *IFI27* has also been shown to discriminate well between influenza and bacterial infections when measured using RT-PCR among people with suspected respiratory tract infection, further supporting its potential clinical utility for the detection of respiratory viruses.^41^

The candidate signatures we evaluated are collectively associated with type I IFN responses, which are a canonical feature of antiviral host defences. The importance of this response in SARS-CoV-2 infection is highlighted by the association of severe COVID-19 with loss-of-function genetic variation in various components of type I IFN pathways and with anti-type I IFN antibodies.^42^, ^43^, ^44^ *IFI27* is best characterised for its functional role in type I IFN-mediated apoptosis as a component of antitumour effects of IFNs.^45^ Differential regulation of IFN-inducible genes might explain why expression of *IFI27* transcripts outperforms other type I IFN signatures and merits further investigation to evaluate the significance of its role in the antiviral response.

A key feature of our study is that all participants self-declared as fit to work when attending study visits, including at the time of their first positive SARS-CoV-2 PCR test, when most participants were asymptomatic. We also found detectable expression of the signatures in blood transcriptomes collected at the study visit 1 week before the first SARS-CoV-2-positive PCR test among a subset of participants. Our data, therefore, show that measurable type I IFN-stimulated responses to SARS-CoV-2 precede the onset of symptoms and, in some individuals, might predate detectable viral RNA on RT-PCR testing. These findings are consistent with previous data showing that transcriptional perturbation preceded symptom onset and detectable viral shedding among a subset of contacts of people with respiratory viral infections.^22^ The point estimate for sensitivity to detect SARS-CoV-2 infection before PCR detection was modest, but larger studies are required to obtain precise performance metrics. We propose that novel diagnostic tests that detect transcripts (or associated protein targets) from the four top-performing candidate signatures we identified could be valuable tools in the rapid detection and isolation of individuals in the very early stages of preclinical infection with SARS-CoV-2. Notably, these signatures also correlated with viral load independently of symptoms, indicating that they have strong potential to identify the most infectious individuals, which is critical to breaking the chains of transmission for SARS-CoV-2.

A key strength of our study was the weekly longitudinal follow-up of study participants, which enabled detailed characterisation of the study cohort, including contemporaneous capture of blood RNA samples at the point of SARS-CoV-2 PCR positivity in pre-symptomatic and asymptomatic infection. We also did a comprehensive systematic literature search to identify candidate blood transcriptional signatures for viral infection. This search enabled direct head-to-head assessments of the signatures' diagnostic accuracy for SARS-CoV-2 infection and will provide a framework for future systematic evaluations of blood transcriptional biomarkers for viral infections.

Our study has some limitations. First, our findings focus on early pre-symptomatic infection and might not be generalisable to moderate or severe COVID-19 disease. Further cohort studies are required to evaluate the diagnostic accuracy of IFN-stimulated host transcriptomic biomarkers for SARS-CoV-2 infection, particularly in the context of more severe disease that could include individuals with attenuated IFN responses—perhaps as a result of host genetics or anti-cytokine antibodies^42^, ^43^, ^44^—and other immunocompromised groups. Second, we did not aim to evaluate discrimination between SARS-CoV-2 and other acute viral infections, and no PCR testing for non-SARS-CoV-2 viruses was done during our study. Because of their discovery in various different viral infections, we expect these 20 signatures to be non-specific biomarkers of acute viral infection. The predictive value of such biomarkers for SARS-CoV-2 infection will be dependent upon pre-test probability, reflecting contemporary transmission rates. Nonetheless, their sensitivity for detecting pre-symptomatic infection offers potential clinical utility for screening contacts of index cases of SARS-CoV-2 to inform infection control management, and stratify the need for confirmatory viral PCR testing. An advantage of non-specific biomarkers of acute viral infection is that their application could extend to acute respiratory viruses generally, and can potentially be multiplexed with prognostic biomarkers. Finally, we intentionally focused the aims of the current study on validation of pre-existing candidate signatures for viral infection for the detection of SARS-CoV-2 to avoid the need for splitting of the cohort for discovery and validation, with a subsequent loss of statistical power. Future studies could consider discovery and validation of SARS-CoV-2-specific signatures when sufficient data become available.

In summary, our findings suggest that a single transcript (*IFI27*) discriminates between individuals with mild early SARS-CoV-2 infection and uninfected healthy individuals with high accuracy. If translated to a near-patient diagnostic test,^46^, ^47^ this transcript could have substantial clinical utility by facilitating early case detection.

## Data sharing

Applications for access to de-identified data for individual participants (including data dictionaries) and samples can be made to the access committee via an online application. Each application will be reviewed, with decisions to approve or reject an application for access made on the basis of accordance with participant consent and alignment to the study objectives; evidence for the capability of the applicant to undertake the specified research; and availability of the requested samples. The use of all samples and data will be restricted to the approved application for access and stipulated in the material and data transfer agreements between participating sites and investigators requesting access. Open access to RNAseq data and associated essential metadata are available under accession number E-MTAB-10022 at ArrayExpress.

## Declaration of interests

We declare no competing interests.
