# Correction to *Lancet Microbe* 2020; published online July 6. https://doi.org/10.1016/S2666-5247(21)00146-4

**DOI:** 10.1016/S2666-5247(21)00185-3

**Published:** 2021-10

*Gupta RK, Rosenheim J, Bell LC, et al. Blood transcriptional biomarkers of acute viral infection for detection of pre-symptomatic SARS-CoV-2 infection: a nested, case-control diagnostic accuracy study.* Lancet Microbe *2021; published online July 6. https://doi.org/10.1016/S2666-5247(21)00146-4*—In this Article, one of Prof R J Boyton's two affiliations was not included. This correction has been made as of July 19, 2021.

