# Supplementary material for: Blood transcriptional biomarkers of acute viral infection for detection of pre-symptomatic SARS-CoV-2 infection: a nested, case-control diagnostic accuracy study
Source: Lancet Microbe. 2021 Oct;2(10):e508–17. doi: 10.1016/S2666-5247(21)00146-4 (PMC8260104; doi:10.1016/S2666-5247(21)00146-4)
Supplement: Supplementary appendix 1 [file mmc1.pdf]

Supplementary data

Figure S1. PRISMA flow-chart of systematic review process for identification of concise transcriptional signatures for the diagnosis of viral infections.. 2

Figure S2. Statistical power and constituent genes comprising four best performing RNA signatures for discriminating participants with contemporaneous PCR-confirmed SARS-CoV-2 infection, compared to uninfected controls.. 3

Figure S3: Peak scores during longitudinal follow-up for best performing RNA signatures to discriminate SARS-CoV-2 cases from heathy controls.. 4

Table S1. Search strategy for systematic Medline search, performed on 12/10/2020.................................. 5

Table S2. Baseline characteristics of the study cohort.. 6

Table S3. Performance metrics of whole-blood RNA signatures whole-blood RNA signatures for discrimination of participants with PCR-confirmed SARS-CoV-2 infection at week prior to first week of PCR-positivity (PCR+ve_-1).. 7

Table S4. Performance metrics of whole-blood RNA signatures for discrimination of participants with PCR-confirmed SARS-CoV-2 infection at week of first week of PCR-positivity (PCR+ve_0) from uninfected controls, excluding participants with contemporaneous symptoms at the time of nasopharyngeal swab sampling. 8

Table S5: Multivariable linear regression model showing associations with IFI27 expression at time of contemporaneous SARS-CoV-2 positivity. 9

Table S6. COVIDsortium investigators (alphabetical order) 10

Figure S1. PRISMA flow-chart of systematic review process for identification of concise transcriptional signatures for the diagnosis of viral infections.

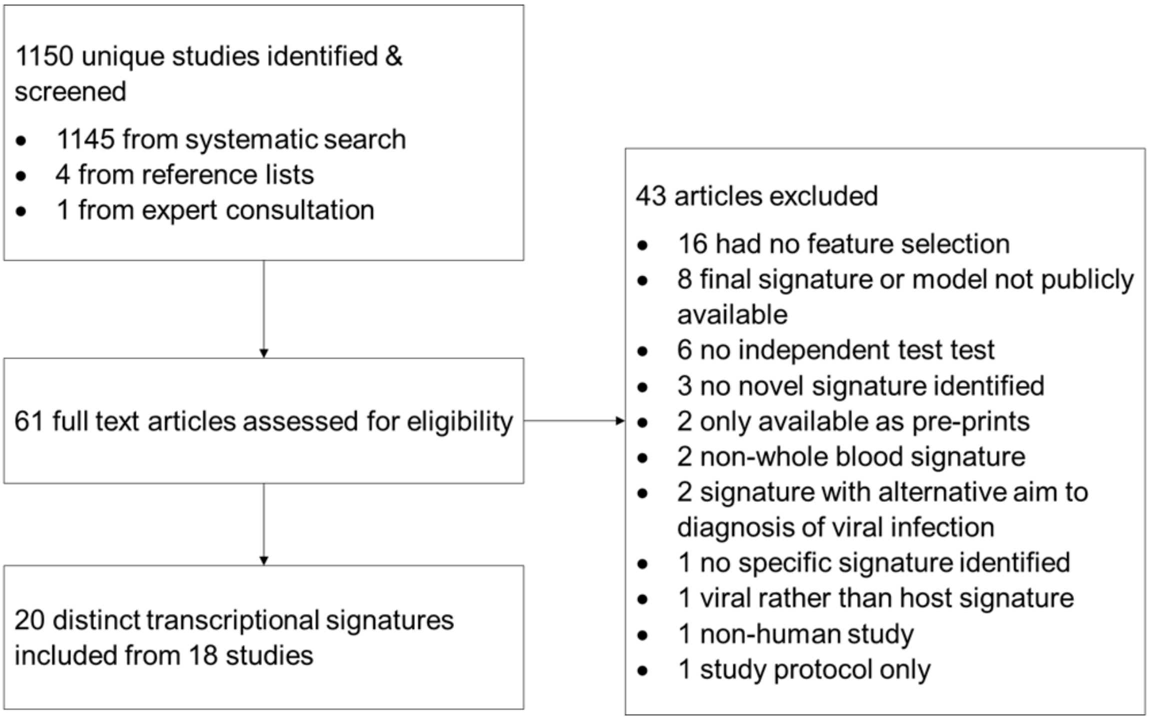

Figure S2. Statistical power and constituent genes comprising four best performing RNA signatures for discriminating participants with contemporaneous PCR-confirmed SARS-CoV-2 infection, compared to uninfected controls.

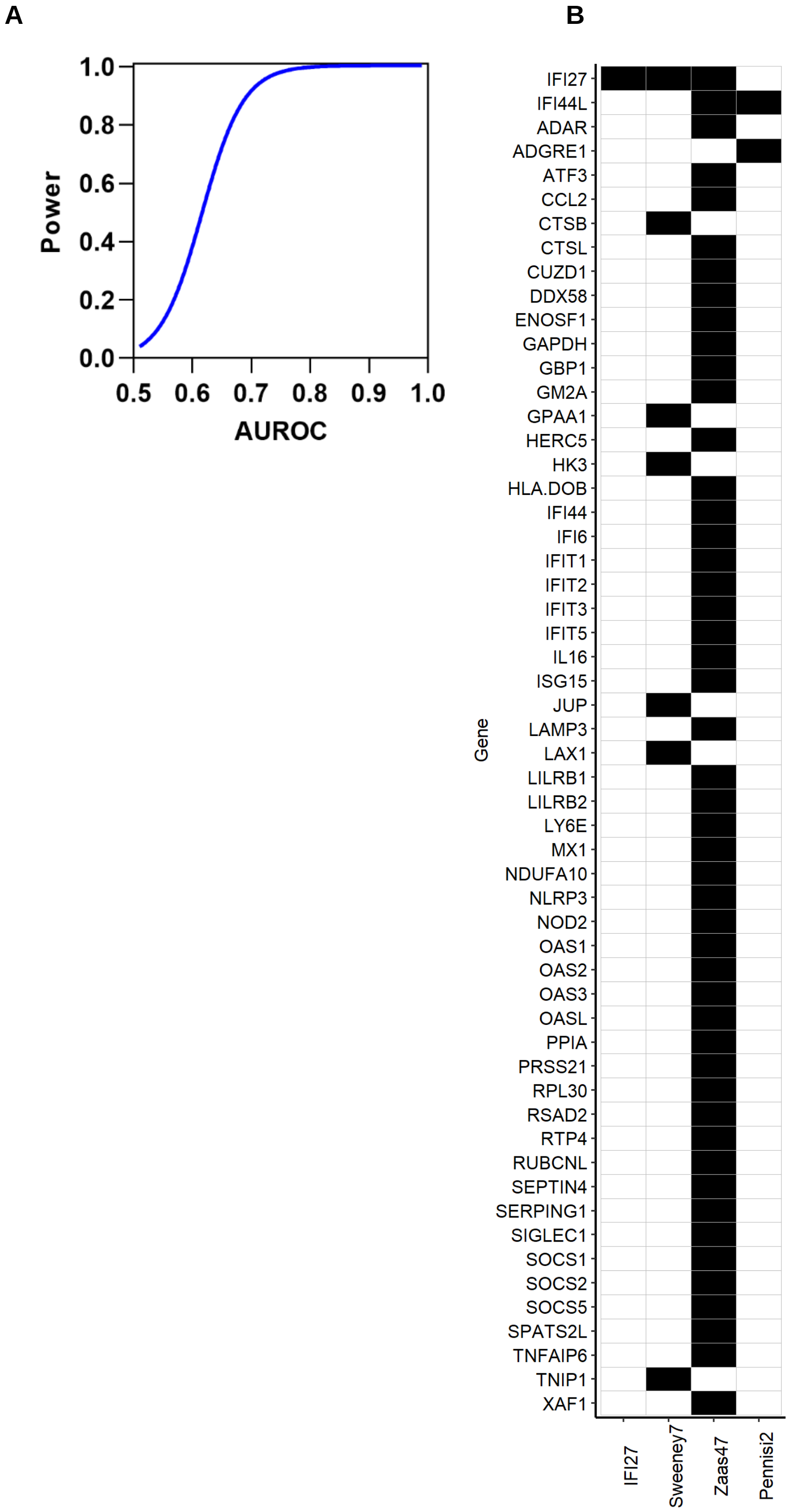

(A) Statistical power to identify discriminate test-positive cases (N=38) from test-controls (N=55) with a given area under the receiver operating characteristic curve (AUROC) with p-value<0.05 (PASS 2021 Power Analysis and Sample Size Software, version 21.0.2, Utah, USA).

(B) Constituent genes (rows) indicated for each signature (columns). Zaas48 includes 49 gene symbols because 2 genes map to a single microarray probe from the original description.

Figure S3: Peak scores during longitudinal follow-up for best performing RNA signatures to discriminate SARS-CoV-2 cases from heathy controls.

AUC = area under the receiver operating characteristic curve (95% CI).

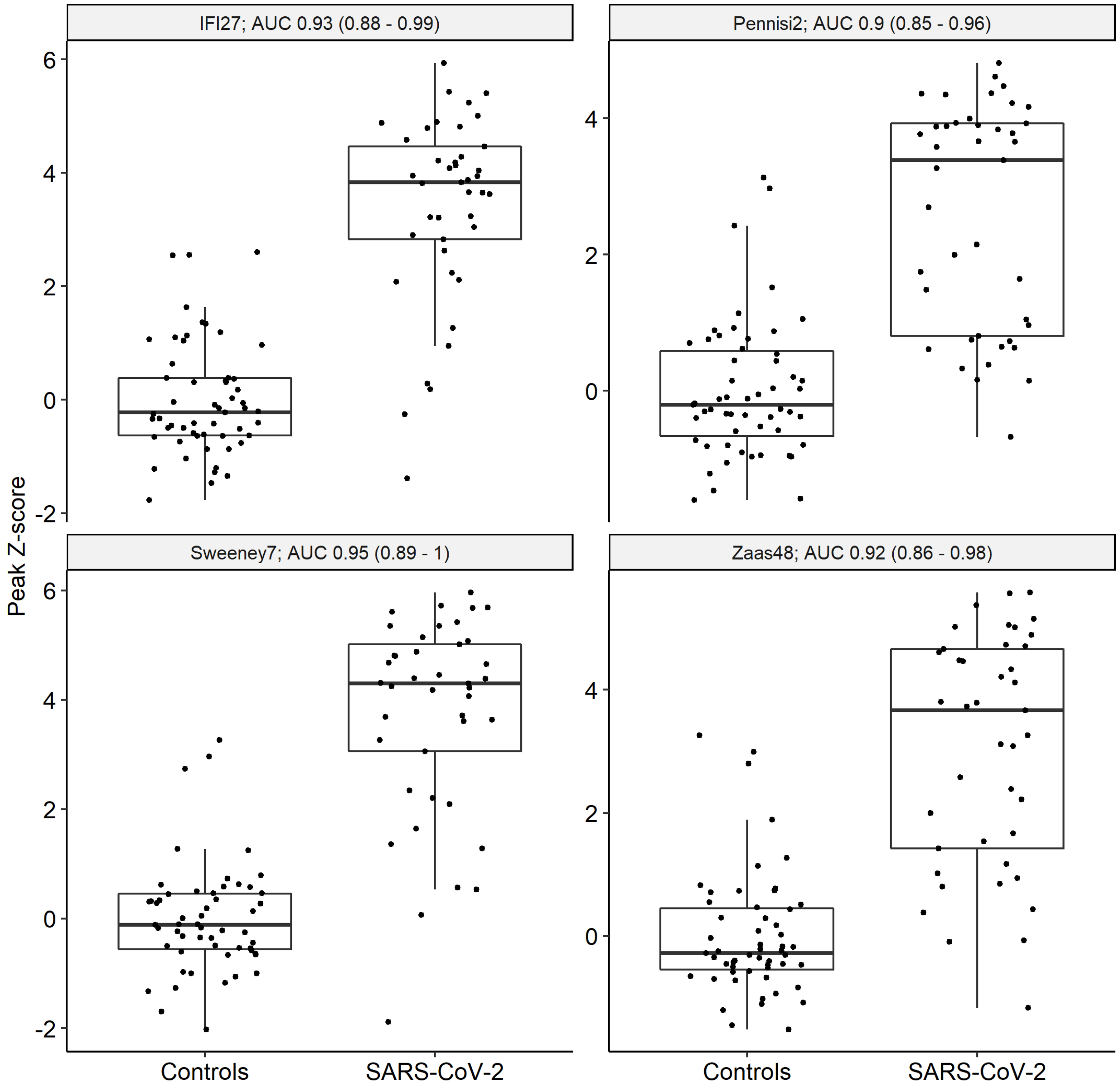

Table S1. Search strategy for systematic Medline search, performed on 12/10/2020.

1. Biomarkers/
2. biomarker*.tw.
3. diagnostic*.tw.
4. signature*.tw.
5. classifier*.tw.
6. 1 or 2 or 3 or 4 or 5
7. Viruses/
8. viral.tw.
9. 7 or 8
10. RNA/
11. Transcriptome/
12. (transcriptom* or transcriptional).tw.
13. (rna or mrna).tw.
14. gene expression.tw.
15. 10 or 11 or 12 or 13 or 14
16. Blood/
17. (host or blood).tw.
18. 16 or 17
19. 6 and 9 and 15 and 18
20. remove duplicates from 19
21. limit 20 to "humans only (removes records about animals)"

Table S2. Baseline characteristics of the study cohort.

Characteristic	Overall, N = 96	Cases, N = 41[1]	Controls, N = 55[1]
Age	36 (27, 47)	36 (28, 46)	36 (26, 50)
Sex			
Female	69 (72%)	28 (68%)	41 (75%)
Male	27 (28%)	13 (32%)	14 (25%)
Ethnicity			
White	66 (69%)	25 (62%)	41 (75%)
Black	6 (6·3%)	5 (12%)	1 (1·8%)
Asian	18 (19%)	9 (22%)	9 (16%)
Other	5 (5·3%)	1 (2·5%)	4 (7·3%)
Unknown	1	1	0
Samples			
1	67 (70%)	12 (29%)	55 (100%)
2	7 (7·3%)	7 (17%)	0 (0%)
3	6 (6·2%)	6 (15%)	0 (0%)
4	10 (10%)	10 (24%)	0 (0%)
5	6 (6·2%)	6 (15%)	0 (0%)
Case-defining symptoms	36 (38%)	31 (76%)	5 (9·1%)

[1]Statistics presented: median (IQR); n (%)

Table S3. Performance metrics of whole-blood RNA signatures whole-blood RNA signatures for discrimination of participants with PCR-confirmed SARS-CoV-2 infection at week prior to first week of PCR-positivity (PCR+ve_-1).

Signature	AUROC	Sensitivity	Specificity
Henrickson16	0·83 (0·71 - 0·96)	0·3 (0·11 - 0·6)	0·93 (0·83 - 0·97)
IFIT3	0·82 (0·69 - 0·94)	0·3 (0·11 - 0·6)	0·93 (0·83 - 0·97)
AndresTerre11	0·82 (0·65 - 0·99)	0·3 (0·11 - 0·6)	0·95 (0·85 - 0·98)
Pennisi2	0·8 (0·64 - 0·95)	0·3 (0·11 - 0·6)	0·95 (0·85 - 0·98)
Zaas48	0·78 (0·6 - 0·97)	0·4 (0·17 - 0·69)	0·95 (0·85 - 0·98)
TrouilletAssant6	0·78 (0·6 - 0·96)	0·3 (0·11 - 0·6)	0·93 (0·83 - 0·97)
IFI27	0·78 (0·59 - 0·97)	0·4 (0·17 - 0·69)	0·95 (0·85 - 0·98)
IFI44L	0·77 (0·6 - 0·95)	0·3 (0·11 - 0·6)	0·95 (0·85 - 0·98)
RSAD2	0·77 (0·6 - 0·95)	0·3 (0·11 - 0·6)	0·93 (0·83 - 0·97)
Sampson4	0·75 (0·58 - 0·93)	0·3 (0·11 - 0·6)	0·93 (0·83 - 0·97)
Sweeney7	0·75 (0·52 - 0·98)	0·4 (0·17 - 0·69)	0·95 (0·85 - 0·98)
Herberg2	0·73 (0·54 - 0·92)	0·3 (0·11 - 0·6)	0·93 (0·83 - 0·97)
Lydon15	0·7 (0·49 - 0·91)	0·3 (0·11 - 0·6)	0·95 (0·85 - 0·98)
Tsalik33	0·68 (0·5 - 0·86)	0·2 (0·06 - 0·51)	0·98 (0·9 - 1)
MX1	0·67 (0·43 - 0·92)	0·3 (0·11 - 0·6)	0·95 (0·85 - 0·98)
Yu3	0·63 (0·44 - 0·83)	0 (0 - 0·28)	1 (0·93 - 1)
Lopez7	0·62 (0·39 - 0·85)	0·2 (0·06 - 0·51)	0·98 (0·9 - 1)
Sampson10	0·57 (0·32 - 0·83)	0·3 (0·11 - 0·6)	0·95 (0·85 - 0·98)
OLFM4	0·49 (0·29 - 0·69)	1 (0·72 - 1)	0·02 (0 - 0·1)
Sweeney11	0·49 (0·26 - 0·72)	0·9 (0·6 - 0·99)	0·04 (0·01 - 0·12)

Includes 10 subsequently SARS-CoV-2 positive participants and 55 uninfected controls. Discrimination is shown as area under the receiver operating characteristic curve (AUROC). Sensitivity and specificity are shown using pre-defined thresholds of 2 standard deviations above the mean of the uninfected control population (Z2). All metrics as shown as point estimates (95% confidence intervals).

Table S4. Performance metrics of whole-blood RNA signatures for discrimination of participants with PCR-confirmed SARS-CoV-2 infection at week of first week of PCR-positivity (PCR+ve_0) from uninfected controls, excluding participants with contemporaneous symptoms at the time of nasopharyngeal swab sampling.

Signature	AUROC	Sensitivity	Specificity
IFI27	0·96 (0·92 - 1)	0·86 (0·69 - 0·95)	0·95 (0·85 - 0·98)
Sweeney7	0·95 (0·9 - 1)	0·86 (0·69 - 0·95)	0·95 (0·85 - 0·98)
Zaas48	0·94 (0·89 - 0·99)	0·62 (0·44 - 0·77)	0·95 (0·85 - 0·98)
Pennisi2	0·92 (0·86 - 0·97)	0·62 (0·44 - 0·77)	0·95 (0·85 - 0·98)
IFI44L	0·91 (0·85 - 0·97)	0·55 (0·38 - 0·72)	0·95 (0·85 - 0·98)
AndresTerre11	0·9 (0·83 - 0·96)	0·55 (0·38 - 0·72)	0·95 (0·85 - 0·98)
Henrickson16	0·89 (0·82 - 0·97)	0·55 (0·38 - 0·72)	0·93 (0·83 - 0·97)
Lydon15	0·88 (0·81 - 0·96)	0·59 (0·41 - 0·74)	0·95 (0·85 - 0·98)
TrouilletAssant6	0·88 (0·8 - 0·95)	0·52 (0·34 - 0·69)	0·93 (0·83 - 0·97)
Herberg2	0·85 (0·77 - 0·94)	0·48 (0·31 - 0·66)	0·93 (0·83 - 0·97)
RSAD2	0·84 (0·75 - 0·93)	0·48 (0·31 - 0·66)	0·93 (0·83 - 0·97)
Sampson4	0·84 (0·75 - 0·93)	0·52 (0·34 - 0·69)	0·93 (0·83 - 0·97)
MX1	0·83 (0·73 - 0·92)	0·45 (0·28 - 0·62)	0·95 (0·85 - 0·98)
Sampson10	0·82 (0·72 - 0·92)	0·52 (0·34 - 0·69)	0·95 (0·85 - 0·98)
Tsalik33	0·8 (0·7 - 0·9)	0·41 (0·26 - 0·59)	0·98 (0·9 - 1)
Lopez7	0·79 (0·69 - 0·9)	0·38 (0·23 - 0·56)	0·98 (0·9 - 1)
IFIT3	0·76 (0·64 - 0·88)	0·45 (0·28 - 0·62)	0·93 (0·83 - 0·97)
OLFM4	0·6 (0·47 - 0·73)	0 (0 - 0·12)	0·98 (0·9 - 1)
Sweeney11	0·57 (0·43 - 0·71)	0·1 (0·04 - 0·26)	0·96 (0·88 - 0·99)
Yu3	0·57 (0·43 - 0·71)	0·07 (0·02 - 0·22)	1 (0·93 - 1)

Includes 29 contemporaneous SARS-CoV-2 positive samples and 55 uninfected controls. Discrimination is shown as area under the receiver operating characteristic curve (AUROC). Sensitivity and specificity are shown using pre-defined thresholds of 2 standard deviations above the mean of the uninfected control population (Z2). All metrics as shown as point estimates (95% confidence intervals).

Table S5: Multivariable linear regression model showing associations with IFI27 expression at time of contemporaneous SARS-CoV-2 positivity.

Includes 31 participants with contemporaneous PCR positivity for SARS-CoV-2. Outcome is IFI27 Z-score.

Characteristic	Beta	95% CI[1]	p-value
(Intercept)	8.4	4.3, 12	<0.001
Cycle threshold	-0.16	-0.26, -0.06	0.003
Age	0.00	-0.05, 0.05	>0.9
Sex			
Female	—	—	
Male	-0.22	-1.4, 0.98	0.7
Current symptoms	-0.39	-1.6, 0.80	0.5

[1]CI = Confidence Interval

Table S6. COVIDsortium investigators (alphabetical order)

Hakam Abbass, Aderonke Abiodun, Mashael Alfarih, Zoe Alldis, Daniel Altmann, Oliver Amin, Mervyn Andiapen, Jessica Artico, João Augusto, Georgiana Baca, Sasha Bailey, Anish Bhuva, Alex Boulter, Ruth Bowles, Rosemary Boyton, Olivia Bracken, Ben O'Brien, Tim Brooks, Natalie Bullock, David Butler, Gabriella Captur, Nicola Champion, Carmen Chan, Aneesh Chandran, David Collier, Jorge Couto de Sousa, Xose Couto-Parada, Teresa Cutino-Moguel, Rhodri Davies, Brooke Douglas, Cecilia Di Genova, Keenan Dieobi-Anene, Mariana Diniz, Anaya Ellis, Karen Feehan, Malcolm Finlay, Marianna Fontana, Nasim Forooghi, Celia Gaier, Joseph Gibbons, Derek Gilroy, Matt Hamblin, Gabrielle Harker, Jacqueline Hewson, Lauren Hickling, Aroon Hingorani, Lee Howes, Alun Hughes, Gemma Hughes, Rebecca Hughes, Ivie Itua, Victor Jardim, Wing-Yiu Jason Lee, Melaniepetra Jensen, Jessica Jones, Meleri Jones, George Joy, Vikas Kapil, Hibba Kurdi, Jonathan Lambourne, Kai-Min Lin, Sarah Louth, Mala Maini, Vineela Mandadapu, Charlotte Manisty, Áine McKnight, Katia Menacho, Celina Mfuko, Oliver Mitchelmore, Christopher Moon, James Moon, Diana Munoz Sandoval, Sam Murray, Mahdad Noursadeghi, Ashley Otter, Corinna Pade, Susana Palma, Ruth Parker, Kush Patel, Babita Pawarova, Steffen Petersen, Brian Piniera, Franziska Pieper, Daniel Pope, Maria Prossora, Lisa Rannigan, Alicja Rapala, Catherine Reynolds, Amy Richards, Matthew Robathan, Joshua Rosenheim, Genine Sambile, Nathalie Schmidt, Amanda Semper, Andreas Seraphim, Mihaela Simion, Angelique Smit, Michelle Sugimoto, Leo Swadling, Stephen Taylor, Nigel Temperton, Stephen Thomas, George Thornton, Thomas Treibel, Art Tucker, Jessry Veerapen, Mohit Vijayakumar, Sophie Welch, Theresa Wodehouse, Lucinda Wynne, Dan Zahedi
